# A True-Color Sensor and Suitable Evaluation Algorithm for Plant Recognition

**DOI:** 10.3390/s17081823

**Published:** 2017-08-08

**Authors:** Oliver Schmittmann, Peter Schulze Lammers

**Affiliations:** Institute of Agricultural Engineering, University Bonn, 53115 Bonn, Germany; lammers@uni-bonn.de

**Keywords:** CIE-Lab, precision plant protection, optical sensor, weed control

## Abstract

Plant-specific herbicide application requires sensor systems for plant recognition and differentiation. A literature review reveals a lack of sensor systems capable of recognizing small weeds in early stages of development (in the two- or four-leaf stage) and crop plants, of making spraying decisions in real time and, in addition, are that are inexpensive and ready for practical use in sprayers. The system described in this work is based on free cascadable and programmable true-color sensors for real-time recognition and identification of individual weed and crop plants. The application of this type of sensor is suitable for municipal areas and farmland with and without crops to perform the site-specific application of herbicides. Initially, databases with reflection properties of plants, natural and artificial backgrounds were created. Crop and weed plants should be recognized by the use of mathematical algorithms and decision models based on these data. They include the characteristic color spectrum, as well as the reflectance characteristics of unvegetated areas and areas with organic material. The CIE-Lab color-space was chosen for color matching because it contains information not only about coloration (a- and b-channel), but also about luminance (L-channel), thus increasing accuracy. Four different decision making algorithms based on different parameters are explained: (i) color similarity (ΔE); (ii) color similarity split in ΔL, Δa and Δb; (iii) a virtual channel ‘d’ and (iv) statistical distribution of the differences of reflection backgrounds and plants. Afterwards, the detection success of the recognition system is described. Furthermore, the minimum weed/plant coverage of the measuring spot was calculated by a mathematical model. Plants with a size of 1–5% of the spot can be recognized, and weeds in the two-leaf stage can be identified with a measuring spot size of 5 cm. By choosing a decision model previously, the detection quality can be increased. Depending on the characteristics of the background, different models are suitable. Finally, the results of field trials on municipal areas (with models of plants), winter wheat fields (with artificial plants) and grassland (with dock) are shown. In each experimental variant, objects and weeds could be recognized.

## 1. Introduction

The use of pesticides in agriculture and green areas is regarded critically. The possible impact of residues on human health and the environment causes decreasing societal acceptance. High costs of pesticides, as well as political regulations call for a reduction of herbicide application in agriculture [1,2]. For the chemical industry, it has become more expensive to develop new agents, but alternative weeding methods have deficiencies, e.g., mechanical methods do not allow weed control on the total crop area and thermal and applications with bio-herbicides are uneconomic [3].

One approach to reduce the amount of pesticides is spot application by decentralized injection of agents into the individual nozzles of a sprayer boom [4,5,6,7,8,9,10]. These treatments can be applied with total herbicides, corresponding selective herbicides, with bio-herbicides or alternative mechanical or thermal measures [11]. However, a precondition is the availability a high-resolution plant recognition system with real-time capability for triggering the actuators. Imaging methods for this purpose do not have this ability yet. The assignment of plants is difficult because plant contours can overlap, and the elapse and response times for real-time processing are not applicable [12].

Conventional RGB color sensors consisting of optical components are real-time capable, but do not have sufficient color accuracy. IR-sensors are not able to distinguish between plant types. High-precision spectrometers are too expensive for agricultural use and are not real-time capable.

The aim of the research is to develop a programmable sensor system, consisting of different single sensors, for the identification of individual crop plants or weeds in municipal and agricultural land and to initiate site-specific treatments. 

Each sensor should cover a small spot of about 20 cm^2^, analyze it and make a decision for or against spraying. The sensor should use the algorithm to develop and switch a valve next to one nozzle in real time. It is expected that small plants, for example in the two-leaf stage, can be detected. The advantage is that small plants can be eliminated with a small dosage very effectively, so that costs and impacts on the environment are reduced.

As a first step, each plant has to be detected. This is sufficient if no differentiation is needed and when unselective treatments are intended (e.g., with glyphosate or bio-herbicides). The second step is to distinguish between crop plants, weeds and plants which can be tolerated.

Smart elements are stated in the fact that each sensor can be programed individually with different decision models for different tasks. Previously, before starting the application, each sensor has to be adjusted. Databases with reflection properties are uploaded on each sensor. Finally, the appropriate decision model and algorithm will be selected and uploaded, as well. 

- Positive recognition:

Spraying is performed if any plant is detected. The reflection properties are within a specified range. Application areas are weed destruction on municipal land or the use as an alternative to glyphosate. 

- Negative recognition: 

No spraying is performed if a crop plant is detected. The reflection properties are out of a specified range. Areas of application are weed destruction in row crops like sugar beet or maize.

- Recognition and differentiation between crops and weeds: 

The range of reflection properties of different plants is compared with the database. Plants are assigned as crop, weed and harmless weed. Application areas are weed destruction on farmland. For example:

○ Arable land with crops like wheat or rape
○ Green areas with grass and broadleaf dock or lawn/golf courses with clover

For this purpose, databases with reflection properties (average and range of values) of different backgrounds (e.g., gravel, stones, soil, grassland) are determined and compiled. Different shades of green, characterizing the spectrum of plant colors, are selected and used for developing algorithms and decision models. These models are based on the color similarity of mixed areas in regard to ΔE, which includes all reflection values, different color and luminance similarity (ΔL, Δa and Δb) and a virtual color channel. The modeling results of the recognition of the greens on different backgrounds are presented.

Additionally, the results of field trials with plants and artificial plants are presented. Exemplarily, the results of the application for plant detection on stones, in winter wheat (*Triticum aestivum* L.) and dock (*Rumex acetosa* L.) in grassland by the use of those algorithms are shown. Finally, the suitability of true-color sensor systems for plant recognition will be evaluated.

## 2. State of the Art

Site-specific plant-protection has been an important field of research over the last few years. Different sensors and methods are used to detect and recognize plants to make a decision for the use of herbicides. In this section, an overview of the state of the art of real-time feasible sensors is presented. It is focused on the suitability for the use in sprayers. Airborne methods or systems that do not refer to single plants or small spots are not taken into account in this overview. The systems can be divided into opto-electronic sensors, imaging techniques and contour-based systems.

### 2.1. Opto-Electronic Sensors

DetectSpray, Weed-Seeker and Green-Seeker are systems for the detection of herbaceous plants on bare soil in reflection mode [13]. The detection principle is based on the fact that green plants absorb red light in the wavelength range between 630 and 660 nm and are highly reflective in the NIR range between 750 and 1200 nm. Basically, in all systems, two monochromatic diodes are used for the R- and the IR-range. For this basic plant identification, the ratio of the R channel to the IR channel is used as a decision criterion [14,15]. When exceeding a threshold value, the existence of plants can be concluded. In contrast to DetectSpray, the Green- and Weed-Seeker [16] devices use an active light source [14].

Approaches about the assignment of indices to plant groups are reported in the literature [17]. For the differentiation of plants, Biller [14] used five sensitive photodiodes with different wavebands to get a ‘spectral fingerprint’ for specific plant types.

Studies on the response accuracy of monochromatic sensors in comparison to real crop plants with weed population showed correlation coefficients of 0.6–0.9 [18]. These systems are described by the Alberta Farm Machinery Research Centre to be negatively affected by sunlight, preventing correct detections. Further, shadowing can hamper the application, especially in row crops [19].

Weed-IT is an Australian system using a sensor scanning a strip of 1 m [20]. It contains an NIR sensor with a light source. According to the manufacturer, it is applicable at high speeds up to 25 km·h^−1^ on stubble fields [12,21]. 

Crop-Cycle (Fa. Holland Scientific, Lincoln, NE, USA) uses reflections in three different wavelengths (670, 730 nm and NIR) for determining the nitrogen supply of plants. Using a calculation model with various indices including a preliminary calibration, the green color can be determined, as well. The manufacturer specifies the size of the measuring area as 20 cm in diameter [22]. 

Finally, traded under the name ‘AmaSpot’, a sensor-nozzle unit, which is based on the Weed-IT system, was awarded as a novelty in 2015 [23].

In these mentioned commercial systems, a 30 × 30 cm^2^ area is scanned, and at least 3% of the scanned surface has to be green for a successful recognition. A scanning area of 50 × 50 cm^2^ needs weeds with a size more than 75 cm^2^. Consequently, either weeds have to be well developed (extended phenotype), or a high degree of coverage by weeds supports a successful recognition and herbicide application [24]. The distinction of plant type and the variation of the scan area size is not possible. Additionally, the system is not ‘open’ to the user. 

Kluge [25] has stated that existing opto-electronic systems are not capable of the distinction between plants, but only generate information on the existence of plants. Therefore, the application of these sensor systems in agriculture is restricted to the period before crops emerge only.

In laboratory experiments, positive results for the determination of plant species by spectrometers are mentioned. Feyaert [26] stated that the differentiation of the reflection characteristics of plant species refers to their physical differences: in the red wavelength by chlorophyll content, which, however, depends on external factors (diseases, water and nutrients), and in the NIR wavelength of the internal structure of the plant, such as cell size and cell wall texture, waxes and trichomes.

### 2.2. Imaging Techniques

Imaging techniques are based on camera systems (CCD camera, bi-, multi- or hyper-spectral) with appropriate optical components and post-processing software. Plant contours can be detected if plants are freestanding [27]. The use of IR-channel shows good results to differentiate between soil and plants [28,29,30]. Imaging techniques are highly sensitive to varying external conditions. Extended computing power is required for weed detection based on shape factors [31,32,33,34]. Overlapping of plant parts makes recognition and plant type differentiation more difficult. 3D camera systems (time-of-flight cameras) actively emit light with a defined wavelength and receive the reflection of the object. They generate real-time images of all three dimensions and additionally a grey-scale image [35]. Time-of-flight cameras are a technical advancement and improve the quality of plant identification, but due to their low resolution and high costs, they are less suitable for practical use.

### 2.3. Plant Identification through Plant Contours without a Camera

Various sensors for plant phenotyping are known. Light grid sensors, consisting of horizontally-cascaded light barriers, were successfully tested [36]. In the described trial, these sensors have been mounted on a carrier vehicle, and measurements have been conducted in a maize field with approximately 20 cm-high plants. Plant identification with light grid sensors in row crops only works for large, non-herbaceous weeds under undisturbed conditions. These sensors are not suitable for narrow-spaced crop recognition. 

Other methods are based on distance sensors (laser and ultrasonic), which determine the contour of the crop plant [37]. Due to the measurement speed, dynamic oscillation of the carrier vehicle and the sensitivity to small changes in distance, this method is not effective [38].

In conclusion, it can be pointed out that the mentioned optoelectronic sensors were well evaluated in former times, but they are not able to detect small plants in early leaf stages (smaller than 3% of the measuring spot). Differentiation of plants seems to be very difficult. Imaging technologies are more complicated, need more computer performance and are too expensive for practical use. Such systems do measure the reflection, but not the real coloration of the object. The identification by the use of plant contours is complicated, as movements of the sprayer or overlapping plants interfere with the measurement of crops. The use of true-color sensors in combination with algorithms is a further development of the presented optoelectronic sensors.

## 3. Material and Methods

### 3.1. Materials

#### 3.1.1. Sensor Technology

Our sensor development is based on the true-color PR0126C sensor of Premosys GmbH (Wiesbaum, Germany). Compared to other sensor systems, true-color sensors represent a compromise between expensive spectrometers with high color accuracy and low cost, but imprecise RGB sensors. The velocity of true-color sensors is much higher than the velocity of spectrometers [39]. The PR0126C sensor can be equipped with different lenses, which determine the spot size of measurement (spot sizes). 

For true-color determination and technical implementation of color standards, true-color sensors are coated with interference filters. Because of this filter characteristics, they are highly capable of color measuring and more sensitive than human eyes (standardized according to the German Institute for Standardization DIN 5033 norm). The sensitivity of the filters is related to a defined spectral wavelength. After normalizing the sensor, the color values are assigned to XYZ coordinates. The XYZ space provides the basis for the conversion into other color spaces. 

The obtained color information then is converted into the CIE-Lab color space. L characterizes the luminance (L: 0 = black, 100 = white). Channels a and b refer to the coloration (a: −128 = green, 127 = red; b: −128 = blue, 127 = yellow). The color space was introduced in 1976 by the International Commission Internationale de l’Eclairage (CIE) and is frequently used by 3D color systems. CIE-Lab is device independent. For plant recognition, the wide green range is a major advantage. 

**Figure 1:**
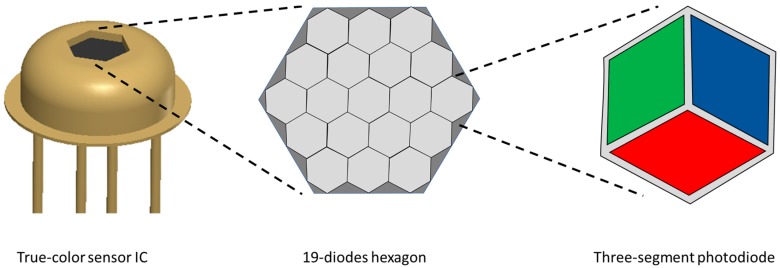
Nineteen-diode hexagon sensor IC with three segment photodiodes for color detection.

The true-color sensor consists of 19-diode hexagon color ICs (integrated circuits, Figure 1) supplied by Mazet GmbH (Jena, Germany) [40]. Each diode has three segments with interference filters for the colors red, green and blue. The components of the sensor are the color-IC, a trans-impedance amplifier, a light-emitting diode with a defined wavelength, a fiber optic for emitting light and receiving reflection and an optical lens. The dimension of the lens (focal length range, measuring spot size and form (point or rod lens)) influences the characteristics of the sensor system in regard to the resolution.

**Figure 2:**
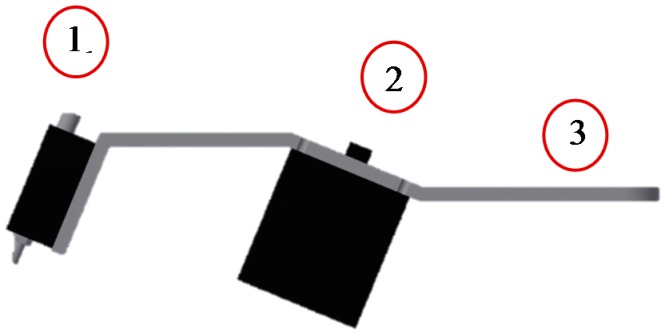
Design of the sensor-lens unit prepared for use in the experiments. Components: 1. True-color sensor with PC interface (RS232), power supply, switching outputs and two optical fibers (emitter and receiver); 2. Lens (double concave, diameter 5 cm, angle 22.5° connector for optical fiber); 3. Space for valve and nozzle.

The sensor was equipped with a double concave lens with a screen diameter of 50 mm (Figure 2). The spot size is about 20 cm^2^.

#### 3.1.2. Objects and Backgrounds

**Figure 3:**
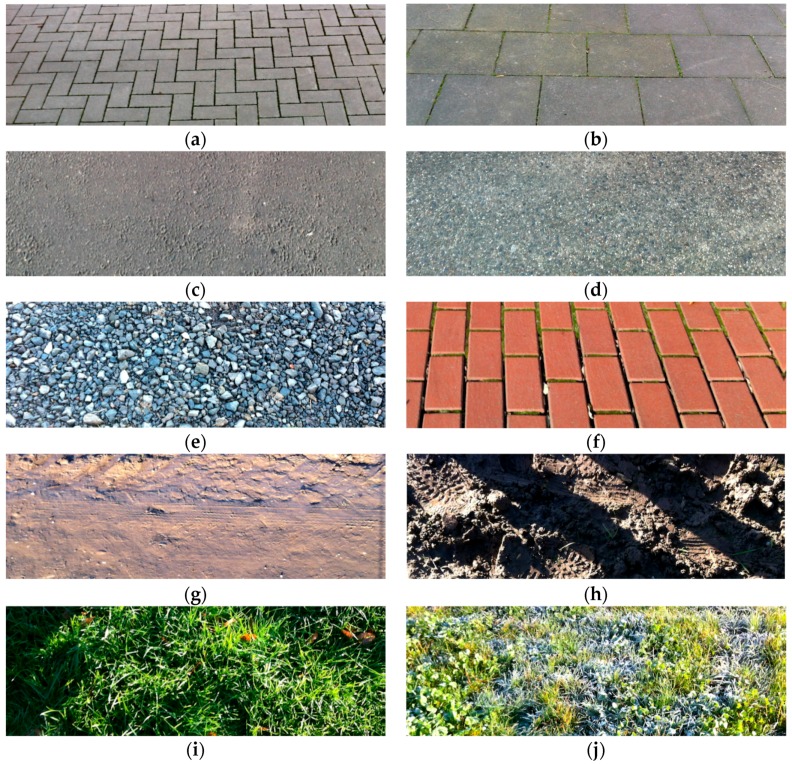
Representation of backgrounds. (**a**) Gray paving stones; (**b**) Stone steps; (**c**) Asphalt; (**d**) Fine chippings; (**e**) Gravel; (**f**) Red paving stones; (**g**) Sandy path; (**h**) Arable land with shadows; (**i**) Grassland; (**j**) Grassland with dew.

For mobile measurements, the backgrounds were divided into anthropogenic and natural. The artificial backgrounds include gravel, concrete slab or paving stones. Natural backgrounds are arable land without vegetation, with stubble or mulch. Green areas were also assigned to this category (grassland with and without dew). The backgrounds are displayed in Figure 3. To characterize different plants by color, four different green color cards from bright to dark green were used.

#### 3.1.3. Test Facilities

**Figure 4:**
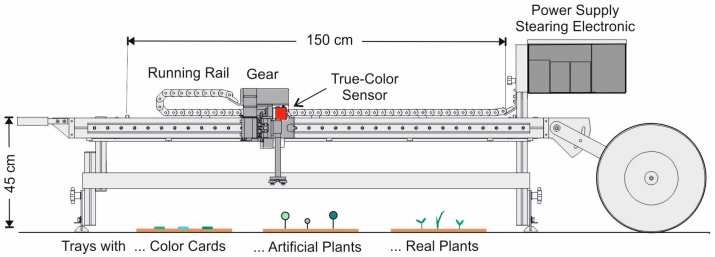
Stationary carrier for dynamic sensor tests.

(a) Stationary Carrier: 

Dynamic measurements on anthropogenic backgrounds were carried out by means of a driven rail system (Figure 4). The sensor was positioned at a predefined height and moved along a distance of 150 cm at a constant forward speed of 0.1 m s^−1^. Different objects, backgrounds and mixed areas were placed below the sensor line. The measuring frequency was 10 Hz (recording of ten color values per second).

**Figure 5:**
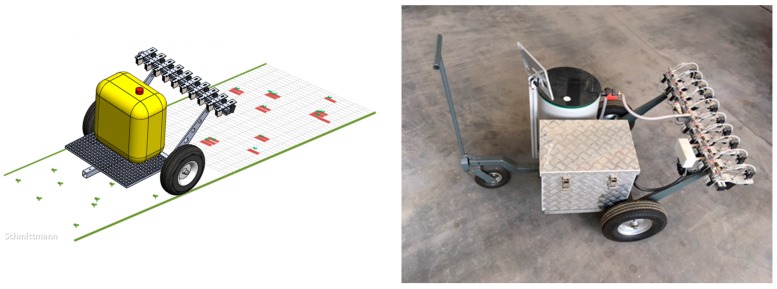
Concept (**Left**) and prototype (**Right**) of the mobile field carrier with spraying device.

(b) Mobile Field Carrier:

For measurements in the field, a mobile carrier was built (Figure 5) for spray application. It can be trailed manually or by a vehicle. Time- and distance-based recordings of color values were performed. 

### 3.2. Methods

#### 3.2.1. Characterization of Backgrounds

In all experiments, objects (plants characterized by cards) and backgrounds (artificial and natural soils with and without vegetation) are characterized by Lab values and their variation (=noise). For this purpose, statistical indicators were used and presented by means of average and frequency distributions. Each background description contains more than 500 values. The backgrounds are divided into human (anthropogenic) and natural vegetation influenced (Figure 5).

#### 3.2.2. Data Management and Processing

Data were collected by means of private domain software. The color values of objects and backgrounds were compiled and implemented into a database, which will be used for decision models and (real-time) plant identification. The setup, justification of the sensors, acquisition, visualization and recording of the data were realized by using this private domain software. However, the software is also important for other aspects, like course-controlled data acquisition, recording of data in a defined format, visualization of measured values and testing of algorithms. 

#### 3.2.3. Decision Making Based on Analyzing the Color Similarity of Mixed Areas

Mixed areas are defined as backgrounds covered with a large or small proportion of objects (plants). The database serves to estimate the potential of plant recognition and identification on different backgrounds. ∆E describes the color distance between two color values in the Lab-color space. According to ISO 12647 [41] and ISO 13655 [42], ∆E for this study is calculated by Equation (1):

(1) $$∆E_{BG,MA}=\sqrt{{\left(L_{BG}-L_{MA}\right)}^{2}+{\left(a_{BG}-a_{MA}\right)}^{2}+{\left(b_{BG}-b_{MA}\right)}^{2}}$$

ΔE_BG,MA_ = color similarity of background and mixed area (background with objects)

L_BG_ = luminance value of background

L_MA_ = luminance value of mixed area

a_BG_ = color value of background for Channel a

a_MA_ = color value of mixed area for Channel a

b_BG_ = color value of background for Channel b

b_MAj_ = color value of mixed area for Channel b

It describes the color similarity between the background without plants and the mixed area: background with plants (Table 1). The range of ΔE is used as a trigger for the decision procedure.

To get more information about color similarity, each channel was analyzed individually. The formulas are given in Equations (2)–(4):

(2) $$∆L_{BG,MA}=\sqrt{{\left(L_{BG}-L_{MA}\right)}^{2}}$$

(3) $$∆a_{BG,MA}=\sqrt{{\left(a_{BG}-a_{MA}\right)}^{2}}$$

(4) $$∆b_{BG,MA}=\sqrt{{\left(b_{BG}-b_{MA}\right)}^{2}}$$

#### 3.2.4. Decision Making Based on Modeling

A statistical model is based on the assumption that the difference of the color values of the background and mixed area should be bigger than the standard deviation of the background without objects and the standard deviation of the object (the sum of both standard deviations (Formula (5)). For decision making, a difference in only one channel may be sufficient. The information about the differences in each channel could be used to classify the plants.

An object exists if:

(5) $$L_{\mathrm{MA}}-L_{\mathrm{BG}}\geq \sigma_{\mathrm{LBG}}+\sigma_{\mathrm{LObj}}\;\vee a_{\mathrm{MA}}-a_{\mathrm{BG}}\geq \sigma_{\mathrm{aBG}}+\sigma_{\mathrm{aObj}}\;\vee b_{\mathrm{MA}}-b_{\mathrm{BG}}\geq \sigma_{\mathrm{bBG}}+\sigma_{\mathrm{bObj}}$$

The required relative coverage area A is calculated as follows:

(6) $$A_{L}=\frac{100\cdot \left(\sigma_{LBG}+\sigma_{LObj}\right)}{\sqrt{{\left(L_{Obj}-L_{BG}\right)}^{2}}}$$

(7) $$A_{a}=\frac{100\cdot \left(\sigma_{aBG}+\sigma_{aObj}\right)}{\sqrt{{\left(a_{Obj}-a_{BG}\right)}^{2}}}$$

(8) $$A_{b}=\frac{100\cdot \left(\sigma_{bBG}+\sigma_{bObj}\right)}{\sqrt{{\left(b_{Obj}-b_{BG}\right)}^{2}}}$$

A_L_, A_a_, A_b_ = minimal relative area with plants covered for Channels L, a and b

L_Obj_, a_Obj_, b_Obj_ = color values of the object for Channels L, a and b

L_BG_, a_BG_, b_BG_ = color values of the background for Channels L, a and b

σ_LObj_, σ_aObj_, σ_bObj_ = standard deviation of the object for Channels L, a and b

σ_LBG_, σ_aBG_, σ_bBG_ = standard deviation of the background for Channels L, a and b

As an additional identification unit, a virtual ‘d channel’ is defined as the difference between the a and b value.

(9) $$d=\sqrt{{\left(a-b\right)}^{2}}$$

## 4. Results and Discussion

### 4.1. Database to Characterize Backgrounds

In Table 2, the reflection values of the anthropogenic and natural backgrounds are listed and characterized by means Ø and standard deviations σ.

- Luminance:

The L-channel in the anthropogenic surfaces is located in a range from 6.39 to 19.31 (Table 2 and Appendix Figure A1). In comparison, the L-channel values of natural backgrounds are in a range from 7.57 to 12.03. Anthropogenic and natural backgrounds are in the same range in this channel. Also important is the variation of these values: it is very conspicuous that the arable land has the smallest standard deviation (0.13). It could be an indicator that even small differences in this channel may be sufficient to detect plants. The biggest deviations are in gravel (1.05, dark and bright stones), red paving stones (1.28, red stone and grey joint) and grassland with dew (2.23).

- Coloration:

Channel a and Channel b give information about the coloration of the backgrounds. Excluding the red paving stones, all tested anthropogenic backgrounds in Channel a are in a range of 0.45–6.52. The range of natural backgrounds is from −8.86 to 5.51. The negative values are caused by the green color of grassland and can be an indicator for the presence of plants. It can be concluded that there is an overlapping of both kinds of background. 

The b-channel is located in the paved surfaces in a range from 10.78 to 25.03 and in the natural backgrounds from 16.16 to 24.80. The range of natural backgrounds is within the range of anthropogenic surfaces.

### 4.2. Characterization of Different Green Tones in Regard to Plant Recognition

In Table 3, the Lab-values for four different green tones are exemplarily listed:

- Luminance:

The variation of the selected greens is higher than the variation of the backgrounds. The range of Channel L is between 29 and 45. In comparison to the backgrounds, the luminance is an outstanding criterion to detect plants. Furthermore, the magnitude of L could be a criterion to differentiate plants and weed. 

- Coloration:

Channel a values are between −15 and −62 and differ in greens significantly from the chosen backgrounds. This wide range of values supports the idea of differentiating different plants by the use of true-color sensors and the CIE-Lab color space. It would be sufficient as the sole criterion for triggering a further evaluation step. In conclusion, the different shades of green can be clearly distinguished by the Lab channels. 

### 4.3. Object Identification by Analyzing Color Similarity

**Figure 6:**
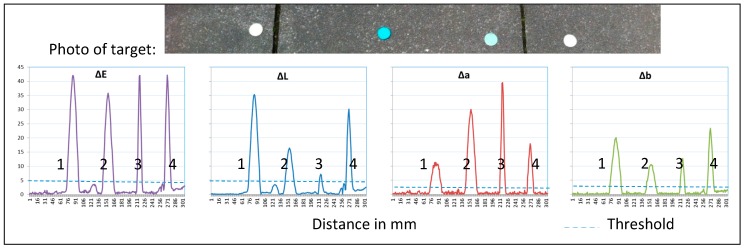
Reflection of paved ground with different colored cards, ∆E, ∆L, ∆a, ∆b and the defined threshold for detection.

The following figures display the described delta values depending on the background characteristics/scattering. For discrimination, a threshold has to be determined. In Figure 6, the delta-signals for solid background are shown exemplarily. This design is the most promising task for weed recognition.

The four objects (r = 1 cm) are detected accurately. The ∆E and ∆L are highly responsive to colored points. By setting a threshold of five, all green objects can be filtered out by the use of ΔE and ΔL. The threshold for Δa and Δb is two. If a measured value is higher, an object/plant exists. To determine the color (kind of plant), the relation of each delta value has to be assessed. The relation between ΔL, Δa and Δb suits the characterization and identification of objects.

**Figure 7:**
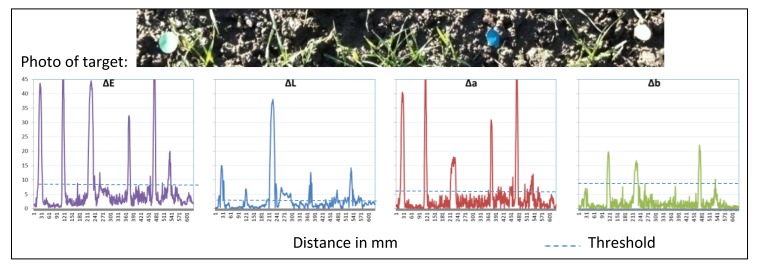
Reflection of a winter wheat field with different colored cards, ∆E, ∆L, ∆a, ∆b and the defined threshold for detection.

The data in Figure 7 are recorded in a young wheat stand (BBCH 13 [44]; Figure 6). Herbicide application at this point in time is common. It is a good example for plant recognition and site-specific spraying in existing crops with selective herbicides. In contrast to the literature [25], it can be shown that the differentiation of plant and weeds is possible by the use of a threshold. With a threshold of 12, all six objects can be identified correctly.

**Figure 8:**
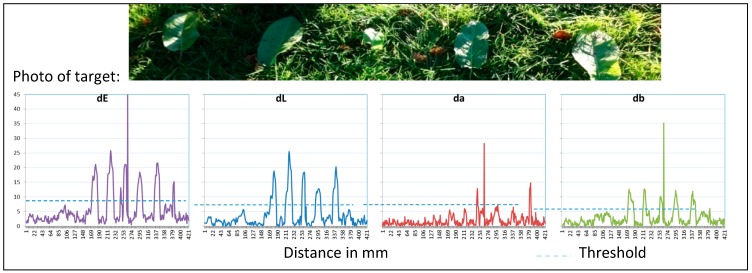
Reflection of grassland with dock, ∆E, ∆L, ∆a, ∆b and the defined threshold for detection.

Figure 8 shows the results of the detection of broadleaf dock on grassland (dock plants are toxic for some animals [45]). A special task is to detect green plants on green areas. ΔE and ΔL are suitable to recognize dock by the use of a threshold of about seven. Δa provides no significant signals. The green color of both plants is quite similar. The reason why the differences in ΔL are higher is caused by the leaf position. A horizontal arrangement of leaves causes higher reflection intensity as the vertical arrangement of leaves from grass. 

### 4.4. Object Identification by Modeling

The result of the decision-based mathematical model (Section 3.2.4) is displayed in Table 4. For each background, the relative object (plant) size of each of the four greens is calculated. Exemplarily, the valuation is displayed for lens diameters of five (spot size 20 cm^2^) and ten centimeters (spot size ~80 cm^2^). 

According to Table 4, it can be concluded that the quality of recognition for all channels is different. As described previously, the influence of the background is relatively low. Green 1 can be identified by the use of the luminance very well. A coverage of 0.5% on the field should be enough for detection. Channel a is also quite suitable, except on red paving stones. 

For Channel L, the sufficient cover of the measurement spot is between 0.5% on fields (Green 1) and up to 13.3% on grassland with dew (Green 4). It is evident that soil without vegetation has the best detection success. The virtual channel d (the difference between a and b) does not show a big advantage. d is more suitable than b, but has no advantage in comparison to a.

## 5. Summary and Conclusions

True-color sensors are a good compromise between inexpensive RGB sensors and spectrometers with high spectral resolution. The objective was to study the suitability of this kind of sensor for the detection and differentiation of crop and weed plants in agricultural and municipal areas. 

For plant-specific spraying, the boom of a sprayer can be equipped with those sensors. The sensor, valve and nozzle together make up an independent sensor-valve-nozzle unit. Depending on sensor spot and spraying angle of the nozzles, the distances between each nozzle can be up to 50 cm. Each true-color sensor is able to control one valve for one nozzle. If spot diameter and nozzle distance are not the same, more sensors (=sensor array) can control the same valve. The possible detection success and identification of small plants is calculated and tested by the use of different algorithms. The spot sizes should be between 20 cm^2^ and 80 cm^2^ to detect plants in the two-leaf stage. The presented detection method contains the following steps:

1. Normalization and adjusting of the sensor in the field. On a place without weeds, the background properties are determined, and a threshold for discrimination will be defined.
2. Selection of the mode: positive or negative detection.
3. Selection and upload of the algorithms and database with reflection properties to each sensor-valve-nozzle unit.
4. Running the plant recognition and differentiation process.
5. Control of the sprayer valve.

The used CIE-Lab color space is suitable for plant recognition, due to the distinction between luminance and coloration. The a-channel (green-red) of the color space is very sensitive for the discrimination of green-colored plants. Databases with reflection properties are assigned. It was shown that backgrounds and green objects are quite different especially in luminance and Channel a.

Four methods for detection are presented:

1. The detection based on color similarity ΔE,
2. The splitting color similarity into ΔL, Δa and Δb,
3. A Virtual Channel d and
4. A modeling algorithm based on statistics.

The suitability of the methods are introduced and have been applied for chosen true backgrounds and different green tones. Methodically, the detection quality and the potential of the sensor were tested under defined conditions on fields with and without vegetation. Based on the experiments carried out, the following statements can be made:

- Due to the built-in single sensor controller, actuators can be addressed and activated in real time.
- A specified evaluation algorithm for plant identification, the decision model and the current calibration values have to be updated to a central computer.
- Minimal differences in coloration (a- and b-channel) and reflectivity can be detected with the sensor. Coloration properties are applicable for plant identification.
- The differentiation of plants in crop and weed appears to be possible by a multistage model. The procedure is as follows:
  1. Extraction of suspicious points by the consideration of ∆E.
  2. Targeted analysis of these signals by the use of the individual Channels L, a and b and
  3. Comparison of all four channels (including Virtual Channel d) relative to each other to decrease the influence of the object size.
- The relative weed/plant coverage of the measuring spot was calculated by a mathematical model. By choosing a decision model previously, the detection quality can be increased. Depending on the background characteristics, different models are more suitable.
- Plants with a size of 1–5% of the measuring spot can be recognized. Weeds in the two-leaf stage can be identified.
- The detection success of the recognition system is displayed and described in field tests. Field trials on municipal areas (with models of plants), winter wheat fields (with models of plants) and grassland (with dock) are shown. In the experiment variants, objects and weeds can be recognized.

It can be stated that true-color sensors are able to detect small differences in luminance and the coloration of objects. They are real-time capable, easy to use and inexpensive. The sensor system is open for the user and can be adapted to the individual condition. In combination with the presented algorithms, it was proven that the sensor has the potential to differentiate between crop and weed. 

In comparison to the existing optoelectronic systems presented in this paper, true-color sensors are further developed. Plants can be detected and discriminated. Even a discrimination of green plants is possible in some cases. An important next step is to evaluate this sensor system under a ‘real field condition’ in different crops. The amounts of savings of herbicides will be the most convincing evaluation parameter. 

## Figures and Tables

**Table 1:** Interpretation and evaluation of the ∆E values ([43], translated).

∆E	Evaluation Categories
0.0...0.5	no to almost no difference
0.5...1.0	difference may be noticeable to the trained eye
1.0...2.0	weak perceptible color difference
2.0...4.0	perceived color difference
4.0...5.0	substantial difference in color, which is rarely tolerated
above 5.0	high difference defined as a different color

**Table 2:** Statistical description of reflection values of selected anthropogenic and natural backgrounds by the mean Ø and standard deviation σ.

	L				a				b			
	Ø	σ	Ø − σ	Ø + σ	Ø	σ	Ø − σ	Ø + σ	Ø	σ	Ø − σ	Ø + σ
**Anthropogenic Backgrounds**												
Grey paving stones	18.69	0.62	18.07	19.31	2.80	1.00	1.80	3.80	20.81	0.54	20.27	21.35
Asphalt	12.91	0.34	12.57	13.25	2.16	1.71	0.45	3.87	13.53	1.12	12.41	14.65
Gravel	7.44	1.05	6.39	8.49	3.15	1.00	2.15	4.15	12.24	1.46	10.78	13.70
Stone steps	13.55	0.50	14.05	14.05	2.34	0.52	1.82	2.86	15.44	0.76	14.68	16.20
Fine chippings	15.55	0.55	15.00	16.10	3.90	0.50	3.40	4.40	18.52	0.84	17.68	19.36
Red paving stones	15.28	1.28	14.00	16.56	13.61	2.89	10.72	16.50	24.12	0.91	23.21	25.03
Sandy path	11.45	0.41	11.04	11.86	4.90	1.62	3.28	6.52	19.72	1.18	18.54	20.90
**Natural Backgrounds**												
Arable land	11.32	0.13	11.19	11.45	4.81	0.70	4.11	5.51	20.10	0.47	19.93	20.57
Grassland	10.35	0.96	9.39	11.31	−2.37	2.40	−4.77	0.03	22.74	2.06	20.68	24.80
Grassland with dew	9.80	2.23	7.57	12.03	−6.32	2.54	−8.86	−3.78	19.47	3.32	16.15	22.79

**Table 3:** Description of the reflection properties of different selected greens.

	Green 1			Green 2			Green 3			Green 4		
	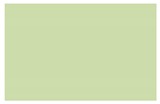			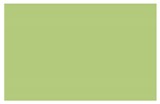			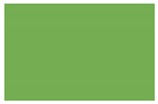			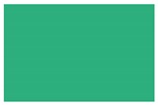		
	L	a	b	L	a	b	L	a	b	L	a	b
Median	44.78	−15.21	44.47	40.45	−26.27	51.75	33.95	−49.86	33.39	29.16	−61.65	−4.33
Mean	44.80	−15.24	44.39	40.45	−26.18	51.80	33.95	−49.95	33.54	29.15	−61.51	−4.22
Minimum	44.71	−14.85	43.79	40.39	−16.83	50.82	32.95	−49.61	32.95	28.98	−63.01	−3.25
Maximum	44.84	−15.73	44.69	40.55	−25.60	52.91	34.47	−50.53	34.47	29.32	−60.35	−4.96

**Table 4:** Required relative coverage of different shades of green on backgrounds for Channels L, a, b and Virtual Channel d.

	Green 1				Green 2				Green 3				Green 4			
	L	a	b	d	L	a	b	d	L	a	b	d	L	a	b	d
Gray pavings	2.5	7.1	3.5	3.0	3.6	7.7	8.5	7.1	5.9	3.6	16.4	5.2	9.2	5.7	6.8	12.2
Asphalt cover	1.2	11.5	4.6	4.4	1.8	10.3	8.4	7.7	2.9	5.0	13.3	5.9	4.2	6.8	12.8	12.3
Gravel	2.9	7.0	5.4	3.5	3.7	7.6	9.0	6.9	5.0	3.6	14.1	5.3	6.4	5.6	15.9	11.0
Flagged floor	1.8	4.6	3.6	3.4	2.5	6.1	7.8	7.1	3.8	2.8	12.7	5.3	5.4	5.0	9.8	11.6
Concrete	2.1	4.1	4.3	2.5	2.9	5.7	8.8	6.6	4.5	2.6	15.9	4.8	6.5	4.8	8.8	10.9
Red pavings	4.5	18.0	5.9	5.6	5.7	15.4	10.9	8.6	8.4	9.2	26.2	6.7	11.7	10.0	7.3	13.4
Sandy path	1.4	9.5	5.9	4.9	2.0	9.1	10.2	8.2	3.1	4.6	19.8	6.3	4.2	6.4	9.8	13.5
Field	0.5	4.9	3.1	2.5	1.0	6.2	8.1	6.6	1.8	3.0	15.0	4.8	2.6	5.1	6.7	8.0
Grassland	2.9	21.0	10.8	8.8	3.7	15.2	14.3	11.5	5.3	7.0	33.6	8.9	6.9	8.5	11.9	20.4
Grassland+dew	6.5	31.8	14.4	15.2	7.8	18.9	16.8	15.6	10.4	7.9	34.8	12.7	13.3	9.4	18.8	25.2
	Spot diameter 5 cm (~20 cm^2^)						...10 cm (~80 cm^2^)									
0–3.0			less:	0.6	cm^2^		less:	2.4	cm^2^	plant shape size						
3.1–5.0				1	cm^2^			4	cm^2^							
5.1–7.5				1.5	cm^2^			6	cm^2^							
7.6–10				2	cm^2^			8	cm^2^							

**Figure A1:**
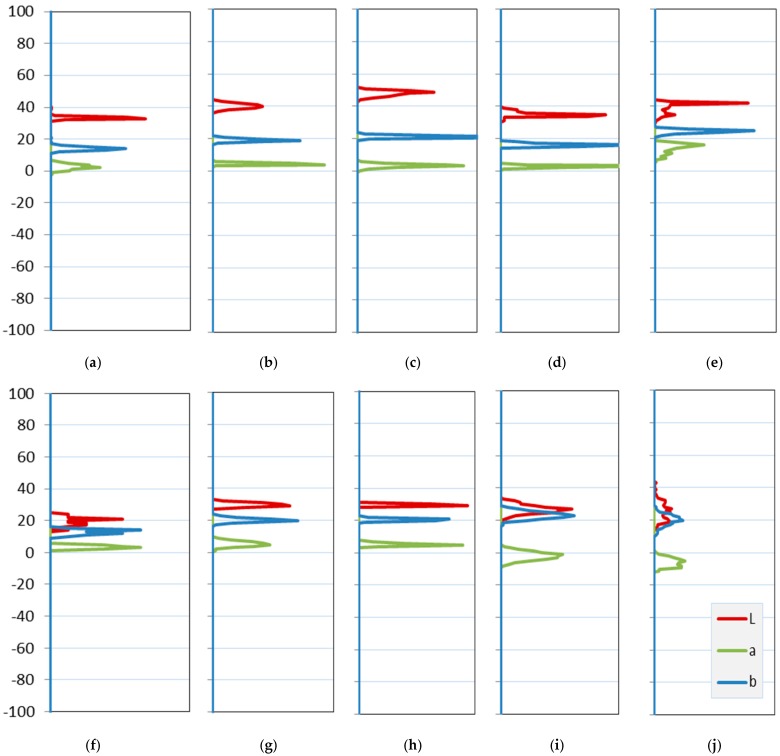
Signal distribution of backgrounds for Channels L, a and b. (**a**) Asphalt; (**b**) Fine chippings; (**c**) Red paving stones; (**d**) Stone steps; (**e**) Gray paving stones; (**f**) Gravel; (**g**) Sandy path; (**h**) Arable land; (**i**) Grazing land; (**j**) Grassland with dew.
